# Hidden in plain sight: urinary *Cryptococcus neoformans* missed by routine diagnostics in a patient with acute leukemia

**DOI:** 10.1186/s12941-022-00540-4

**Published:** 2022-11-12

**Authors:** Zoe F. Weiss, James E. DiCarlo, David W. Basta, Stephanie Kent, Alexis Liakos, Lindsey Baden, Manfred Brigl, Sanjat Kanjilal, Connie Cañete-Gibas, Nathan P. Wiederhold, Sankha S. Basu

**Affiliations:** 1 Department of Pathology, Brigham & Women’s Hospital and Harvard Medical School, Boston, MA USA; 2 Division of Infectious Diseases, Brigham & Women’s Hospital and Harvard Medical School, Boston, MA USA; 3 Department of Pathology, Tufts Medical Center, Boston, MA USA; 4 Department of Population Medicine, Harvard Pilgrim Healthcare Institute and Harvard Medical School, Boston, MA USA; 5 Fungus Testing Laboratory, Department of Pathology and Laboratory Medicine, Long School of Medicine, University of Texas Health San Antonio, Texas, USA

**Keywords:** Cryptoccouria, *Cryptococcus neoformans*, Cryptococcal infection, MALDI-TOF MS, India-ink, Cryptococcal antigen

## Abstract

Cryptococcuria is a rare manifestation of localized cryptococcal disease. We present a case of *Cryptococcus neoformans* urinary tract infection in an immunocompromised host missed by routine laboratory workup. The patient had negative blood cultures, a negative serum cryptococcal antigen (CrAg), and “non-*Candida* yeast” growing in urine culture that was initially dismissed as non-pathogenic. The diagnosis was ultimately made by matrix-assisted laser desorption/ionization time-of-flight mass spectrometry (MALDI-TOF MS) from a repeat urine culture after transfer to a tertiary care center. *Cryptococcus* should be considered in the differential of refractory urinary tract infections growing non-*Candida* yeast.

**Supplementary Information:**

The online version contains supplementary material available at 10.1186/s12941-022-00540-4.

## Background

*Cryptococcus neoformans* is a free living saprobic encapsulated yeast that is ubiquitous in the environment and typically causes infection in patients with immunocompromising conditions such as HIV, hematologic malignancy, or organ transplantation. The cryptococcal polysaccharide capsule is its main virulence factor, allowing it to evade host immunity and readily disseminate. Once inhaled, pulmonary infection can lead to hematogenous spread, most often to the central nervous system but occasionally to skin, bone, and rarely the prostate or bladder. Cryptococcuria is a rare clinical finding. One retrospective study in an 800-bed hospital over 12 years found an incidence of 0.56 patients per 100,000 [1]**.**

Identification of *C. neoformans* growing from clinical samples traditionally involves routine microbiology workup including specific staining techniques along with selective and differential media. Though colony morphology is not a specific diagnostic feature, *C. neoformans* is typically opaque to white and mucoid appearing on blood agar, while strains lacking capsules may appear dry [2, 3]. Increasingly, the use of proteomic techniques such as MALDI-TOF MS and DNA sequencing of the internal transcribed spacer (ITS) region of the nuclear ribosomal DNA have replaced traditional phenotypic methods for identification from culture [4]. Additionally, the use of highly sensitive and specific serum and cerebrospinal fluid (CSF) lateral flow antigen assays to detect Cryptococcal capsular antigens from clinical samples have become important non-culture based diagnostic tools. There are no FDA approved CrAg assays for urinary samples. *Cryptococcus* may be missed from urine in laboratories that do not routinely identify all yeasts in urine cultures, as yeast is frequently a commensal or non-pathogenic organism in urine samples, particularly in patients with indwelling Foley catheters. Here we describe a case of *C. neoformans* isolated only from urine identified initially by MALDI-TOF MS.

## Case

A 77-year-old Caucasian man with a long-standing history of high-risk myelodysplastic syndrome with systemic mastocytosis and benign prostatic hyperplasia was admitted to an outside hospital in the Boston (USA) area with several weeks of fatigue, somnolence, weakness, weight loss, and worsening nocturia. Two weeks prior to his hospitalization, he reported receiving cephalexin at a local clinic for a urinary tract infection (no culture data available), however he continued to experience urinary frequency and pain. He had no recent or remote travel outside of the New England area.

On presentation he was hypothermic (31 °C), bradycardic (HR 40 bpm), and hypotensive (systolic blood pressure was 80 s-90 mmHg). His laboratory workup was notable for hypoglycemia (glucose 22 mg/dL), a creatinine of 1.5 mg/dL (baseline 1.4 mg/dL), and evidence of malignant transformation to acute myeloid leukemia (AML). His white blood cell (WBC) count was 57,000 cells/mcL with 23% blasts. Peripheral flow cytometry revealed new acute AML with myelomonocytic differentiation. His urinalysis revealed moderate leukocyte esterase, > 50 WBCs per high powered field, and moderate budding yeast identified as non-*Candida albicans* yeast by germ tube test without further identification due to laboratory protocol. Sputum and blood cultures taken on admission were negative. Urine antigen testing for *Streptococcus pneumoniae* and *Legionella* were negative.

CT scan of the chest showed consolidative opacities in the left upper and right lower lobes concerning for multifocal pneumonia. CT scan of the abdomen showed stable known left hydronephrosis. He was started on empiric vancomycin and ceftazidime for a urinary tract infection and presumed bacterial pneumonia. A Foley catheter was placed. His course was complicated by disseminated intravascular coagulation, acute kidney injury, and tumor lysis syndrome for which he was stabilized with transfusions, rasburicase, and hydroxyurea. He was transferred to Brigham and Women’s Hospital in Boston, MA on day 11 for ongoing management of his AML. Repeat urine samples were not immediately obtained.

**Fig. 1:**
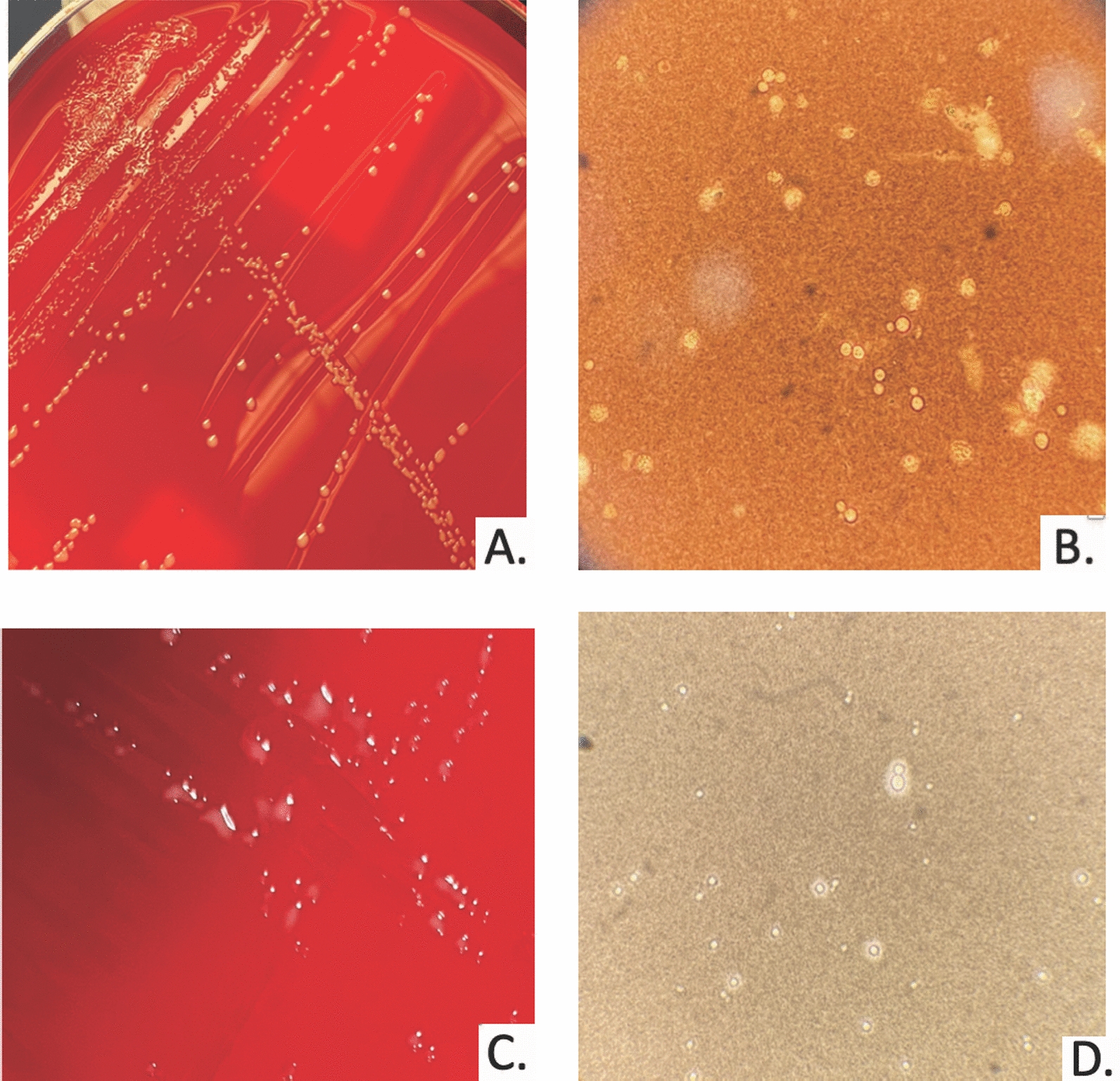
Clinical isolate on sheep's blood agar and India ink staining. **A** Urine culture on Sheep's Blood agar. *C. neoformans* colonies appear white, dry, and non-mucoid. **B** Cultured isolate by microscopy, no encapsulated yeast seen by India ink preparation. **C** Urine culture on Sheep's Blood agar after four days of antifungal therapy. Raised white mucoid colonies are morphologically consistent with *C. neoformans.* **D** Cultured isolate after four days of antifungal therapy, encapsulated yeast seen on India ink preparation

On day 26, the patient was noted to be transiently somnolent with no other localizing symptoms. His WBC count was 2690 cells/mcL with an absolute neutrophil count of 960 cells/mcL and absolute lymphocyte count of 750 cells/mcL. His creatinine was 2.1 mg/dL. Repeat blood and sputum cultures were negative. Urinalysis showed moderate leukocyte esterase, negative nitrites and 135 WBCs per high powered field. The urine culture grew > 100,000 colonies forming units (CFU) of yeast, which appeared as uniformly dry white colonies (Fig. 1A). Our laboratory protocol requires yeast species identification if the organism is predominant and > 50,000 CFU. Using matrix-assisted laser desorption/ionization time-of-flight mass spectrometry (MALDI-TOF MS, VITEK MS, Biomerieux), the organism was identified as *Cryptococcus neoformans* with > 99.9% confidence on two different instruments. An India ink stain of the urine culture isolate was requested for further confirmation of this unexpected finding and was negative (Fig. 1B). Serum CrAg (IMMY, Crypto-Latex Antigen Detection System; Immunomycologics Inc., Norman, OK, USA) was negative on serial dilution. ITS, D1/D2 sequencing and susceptibility were performed at Fungus Testing Laboratory, University of Texas Health at San Antonio, San Antonio Texas and again confirmed as *Cryptococcus neoformans var. grubii (*VNI/ VNII) (see Additional file 1: Fig. S1) [5]. Susceptibility by broth microdilution revealed a fluconazole MIC of 4 mcg/ml.

Given negative blood cultures, negative serum antigen testing, and no neurologic symptoms (no headaches, meningeal signs, fevers), and thrombocytopenia, lumbar puncture and induction with amphotericin were deferred. The patient was started on oral fluconazole 800 mg once, then 400 mg daily for treatment of isolated cryptococcuria in the absence of evidence of disseminated disease. On day 30, four days into antifungal therapy, a repeat urine sample demonstrated yeast which grew with a more typical mucoid appearance for *Cryptococcus* (Fig. 1C), which was again confirmed as *C. neoformans* by MALDI-TOF MS and now stained positive by India ink (Fig. 1D). Serum CrAg remained negative. On day 35 the patient was discharged on fluconazole indefinitely. His urologic symptoms resolved with fluconazole therapy, although he sadly expired two months after discharge due to complications from AML, unrelated to cryptococcal infection.

## Discussion

The cryptococcal capsule, largely composed of the polysaccharides glucuronoxylomannan and glucuronoxylomannogalactan, is an important virulence factor for infection and can morph in size depending on environmental pressures exerted by the host or artificial growth media. In vivo, the protective capsule allows for immune evasion through prevention of phagocytosis [2, 6] and numerous immunomodulating properties, including downregulation of pro-inflammatory cytokines and complement depletion. Phenotypic switching and differential capsular expression are associated with changes in virulence, with unencapsulated cells being relatively avirulent and rarely associated with clinical isolates [7]. In vitro, decreased expression of capsular phenotypes in the absence of environmental pressure may result in variable culture morphologies depending on growth conditions [3]**.**

India ink is traditionally used to detect cryptococcal organisms in clinical specimens by revealing the polysaccharide capsule which appears as a "halo" around the organism due to the inability of the ink to penetrate the capsule (Fig. 1D). However, poor sensitivity in clinical samples [8] and morphological variability in culture media limits its utility [2, 3]. Use beyond CSF samples is not established. Case reports describe its use in urine samples [9].

The initial isolate from the urine culture for our patient appeared dry and non-mucoid, consistent with a lack of visible capsules on India ink preparation (Fig. 1A and B). Repeat urine culture while on antifungal therapy revealed an isolate that appeared mucoid and was India ink positive. Capsular expression is regulated by complex host environmental cues. The initial morphology may have been due to a reduced capsular expression in vitro versus selective isolation of a phenotypically capsule deficient strain at the time of collection. Antifungal therapy is also associated with modulation of capsule size while specific host immune responses can induce or select for a larger capsule [10, 11]. It is possible that the distinct change in morphology was related to the host response to treatment initiation.

**Fig. 2:**
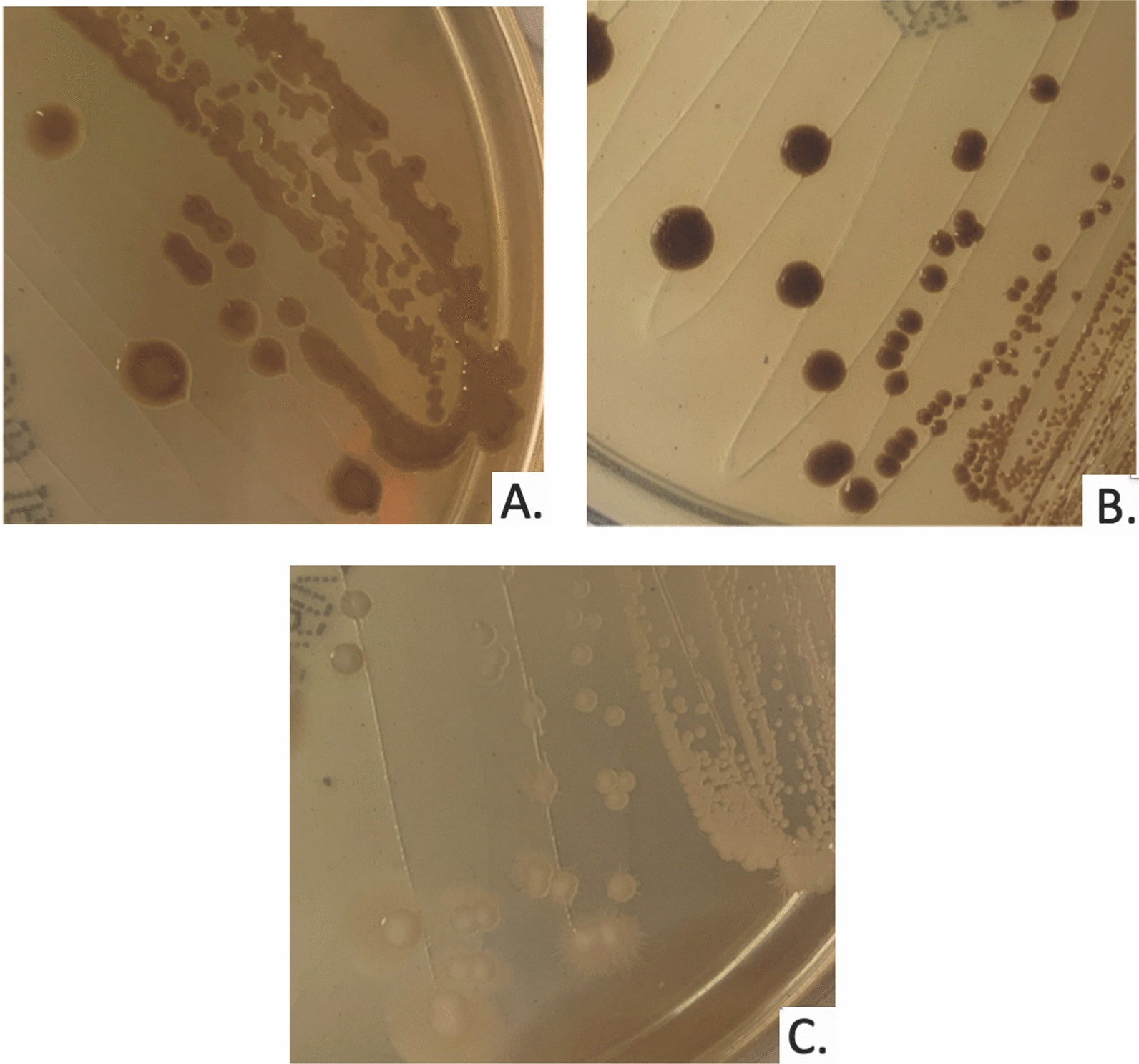
*Cryptococcus neoformans* and *Candida albicans* on Niger seed extract agar after 5 days of growth. **A** *C. neoformans* ATCC 14,116. **B** Clinical isolate *C. neoformans*. **C** *C. albicans* ATCC 14,053

Identification of *Cryptococcus* in this case was achieved through urine culture plated to solid media followed by MALDI-TOF MS. Additional confirmation was sought using ITS and D1/D2 sequencing. In general, samples plated on 5% Sheep's Blood agar or Sabouraud dextrose agar can readily grow *Cryptococcus* sp. at 37 °C as early as 1–2 days after culture inoculation, however some isolates may take up to 5 days [12]. Historically, selective and differential media, were used to identify *Cryptococcus* sp. Compared to *Candida sp.*, which remain white, *Cryptococcal* colonies appear dark brown on agar containing Niger seed extract (Fig. 2). Further differentiation of *C. neoformans* and *C. gattii* can be accomplished using L-Canavanine glycine bromothymol blue containing media, with C. gattii metabolizing the glycine, increasing the pH and turning the colonies blue via the bromothymol blue indicator [13].

Less labor-intensive phenotypic methods have subsequently emerged. Biochemical assays, such as those employed by the API 20C AUX test strip by bioMerieux are an essential part of yeast identification in many microbiology labs. The use of MALDI-TOF MS for rapid proteomic identification of *Cryptococcus* from cultured isolates has increasingly replaced traditional phenotypic techniques with a high degree of accuracy, especially for the most common species of *Cryptococcus* (*C. neoformans* and *C. gattii*). Further improvements in commercial database curations are still required to discriminate among less common species [4]. Though sequencing remains the gold standard for species identification, costs and turnaround time limit its use in the routine clinical laboratory.

Given the limitations of microscopy, reduced availability and prolonged turnaround time for culture-based methods, clinicians world-wide still rely heavily on the use of rapid, point of care, lateral flow assays (LFA) to quickly and accurately detect *Cryptococcus* antigen in serum and cerebrospinal (CSF). These dipstick tests use monoclonal antibodies embedded on an immunochromatographic test strip to signal the presence of cryptococcal capsular polysaccharide glucuronoxylomannan antigen. In a meta-analysis of 12 studies (primarily of HIV-positive individuals) Huang et al. reported the pooled sensitivity and specificity of CSF and serum CrAg to be ~ 98–99% respectively for cryptococcosis [14]. Serum cryptococcal antigen may be less sensitive, however, for localized forms of the disease [15] and other specimen sources (such as from urine) have not been well established or FDA approved. Huang et al. reported a sensitivity of 85% from urine CrAg with insufficient information for specificity calculation [14]. The poor performance of urine CrAg precludes its current use in routine laboratories [16].

A negative serum CrAg may be due to localized disease due to reduced sensitivity in this setting. A negative CrAg LFA may also occur in the setting of capsule deficient, or capsule reduced organisms, as this method is specific for the polysaccharide *Cryptococcus* capsule. Though this is a rare phenomenon it has been described in the literature [17]. False negative antigen testing can occur in two additional scenarios. In cases of overwhelming cryptococcosis and appropriate immune response, high levels of endogenous antibody may sequester free antigen and thus interfere with antigen testing, called the “Prozone Phenomenon”. Alternatively, the “Post-Zone Phenomenon” may also occur where excessive antigen relative to immunoassay antibodies prevent antibody-antigen cross linking required for immunochromatographic detection [18].

Our patient presenting with new AML had over a month of urological symptoms, an abnormal urinalysis, multiple negative routine blood and sputum cultures, negative serum CrAg, and urine cultures growing non-*Candida* yeast ultimately identified as C*. neoformans*. Cryptoccocuria is a rare clinical entity that is typically associated with systemic cryptococcal disease and has been described in both immunocompromised and in immunocompetent hosts [19]. In one retrospective analysis of 58 cases of cryptococcuria, 46 (79.3%) had a CrAg positive in CSF and 39 (67.2%) in blood. In 8 cases, cryptococcuria preceded the diagnosis of disseminated cryptococcosis. As such, identification in the urine may be an early indication of disseminated disease [1, 20]. Cryptococcuria has also been described in cases of chronic prostatitis diagnosed by prostatic biopsy and may represent a potential reservoir for infection in males [21, 22]. Cases of pyelonephritis [23] and epididymitis [24] have also been reported.

We demonstrate a rare case of urinary *C. neoformans* initially missed due to a laboratory protocol that precluded the workup of non-*Candida* yeasts from urine and lack of clinical suspicion. The microscopically observed variability in colony morphology and India ink staining may be attributed to variable in vitro growth conditions, the presence of multiple strains, or possibly altered capsular expression in the setting addition of antifungal agents and subsequent immune response. India ink is not routinely used in clinical samples other than CSF and urine microscopy is not sensitive for this organism. Urine cryptococcal antigen testing is not recommended and serum antigen testing may be negative in localized disease or rarely in the setting of capsule deficient strains. False negatives may also occur due to both the “Prozone Phenomenon” and “Post-Zone Phenomenon” and clinicians should understand the limitations of antigen testing. Proteomic techniques, which are increasingly being incorporated into clinical laboratories, can reliably confirm the diagnosis from pure culture.

Cryptococcuria is a rare clinical entity and can be associated with systemic disease or a harbinger for systemic involvement as described in the literature. Clinicians should consider *Cryptococcus* in cases of treatment-refractory urinary tract infections with non-*Candida* yeast in immunocompromised hosts, even when serum antigen testing and blood cultures are negative. In these cases, clinicians should request further workup of non-*Candida* yeasts from urine if not routinely performed.

## Supplementary Information

**Additional file 1: Figure S1.** Maximum likelihood tree resulting from ITS sequence analysis of isolate UTHSCSA DI22-46 and sequences of representative strains of the *Cryptococcus neoformans* species complex and the *Cryptococcus gattii* species complex obtained from GenBank. Confidence values at the nodes > 0.95 [25, 26] represent posterior probabilities from Bayesian analysis, > 80% represent bootstrap re-samplings. The scale bar shows expected number of changes per site. The maximum likelihood analysis was conducted in IQ-Tree using the substitution model TIM + F determined by corrected Akaike Information Criteria (AIC) and Model Finder both of which are implemented in IQ-Tree [27–29].

## Data Availability

All data generated or analyzed during this study are included in this published article [and its supplementary information files].

## Abbreviations

MALDI-TOF MS: Matrix-assisted laser desorption/ionization time-of-flight mass spectrometry
AML: Acute myeloid leukemia
WBC: White blood cell
CrAg: Cryptococcal antigen
ITS: Internal transcribed spacer
CSF: Cerebrospinal fluid
LFA: Lateral flow assay
