# Supplementary figures and images for: Hidden in plain sight: urinary Cryptococcus neoformans missed by routine diagnostics in a patient with acute leukemia
Source: Ann Clin Microbiol Antimicrob. 2022 Nov 12;21:49. doi: 10.1186/s12941-022-00540-4 (PMC9655867; doi:10.1186/s12941-022-00540-4)

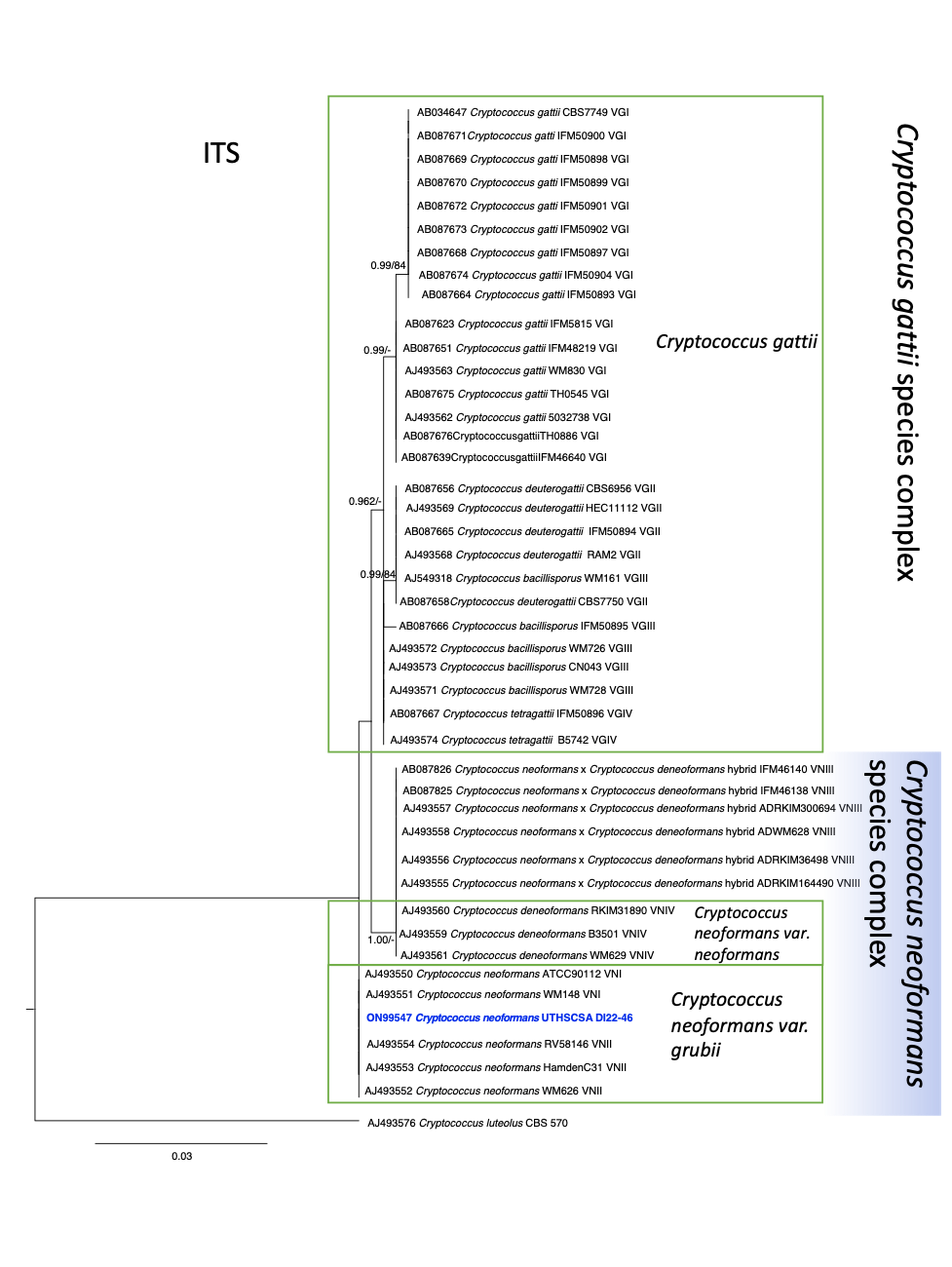

Supplement: Supplementary file 1 — Additional file 1: Figure S1. Maximum likelihood tree resulting from ITS sequence analysis of isolate UTHSCSA DI22-46 and sequences of representative strains of the Cryptococcus neoformans species complex and the Cryptococcus gattii species complex obtained from GenBank. Confidence values at the nodes > 0.95 [25, 26] represent posterior probabilities from Bayesian analysis, > 80% represent bootstrap re-samplings. The scale bar shows expected number of changes per site. The maximum likelihood analysis was conducted in IQ-Tree using the substitution model TIM + F determined by corrected Akaike Information Criteria (AIC) and Model Finder both of which are implemented in IQ-Tree [27–29]. [file 12941_2022_540_MOESM1_ESM.png]
